# Pathological gambling in a patient on piribedil: A case report

**DOI:** 10.1097/MD.0000000000024568

**Published:** 2021-02-12

**Authors:** Yingtan Wang, Zhe Lu, Guanglei Xun

**Affiliations:** a Department of Mental Health, Jining Medical University, Jining; b Peking University the Sixth Hospital (Institute of Mental Health), Beijing; c Shandong Mental Health Center, Jinan, China.

**Keywords:** case report, depressive disorder, dopamine partial agonist, gambling disorder, Parkinson disease, pathological gambling, piribedil

## Abstract

**Rationale:**

Piribedil is an orally active dopamine agonist that has been widely used for Parkinson disease (PD), with its partial D2/D3 agonistic functions and alpha2-adrenoreceptor antagonistic effects, piribedil has been proved to be efficacious in the relief of motor symptoms in PD, while it can also lead to impulse control disorders such as pathological gambling due to its dopamine agonistic effects.

**Patient concerns:**

A 28-year-old Chinese female patient with Parkinson disease and a history of taking piribedil finally developed pathological gambling and depressive episode.

**Diagnoses:**

After a careful clinical observation and evaluation, the patient met the criteria of severe depressive episode and pathological gambling due to antiparkinson therapy.

**Interventions:**

We discontinued piribedil and picked bupropion, a dopamine reuptake inhibitor, to alleviate the depressive symptom. Benzhexol and selegiline were also added for the control of motor fluctuations.

**Outcomes:**

After 3 weeks’ treatment, the patient's depressive mood was significantly alleviated and her recurring PD symptoms were also relieved. She was no more addicted to network gambling, and there was no recurrence during the 1-year follow-up.

**Lessons:**

Piribedil-induced problem gambling and impulse control disorders are side effects needed to be evaluated when commencing a patient on piribedil. This case further emphasizes the importance of monitoring and controlling Parkinson symptoms after drug reduction or withdrawal. Anticipation of this risk strengthens the significance of detailed medical history-taking and targeted clinical management.

## 1 Introduction

Pathological gambling (PG) was originally included as a mental health diagnosis in 1980 in the Diagnostic and Statistical Manual of Mental Disorders (DSM).^[1]^ According to the fifth edition of the DSM (2013), it was grouped together with substance-related and addictive disorders, and was renamed to gambling disorder (GD), which was defined as a persistent and recurrent problematic gambling behavior leading to clinically significant impairment or distress.^[2]^ In the latest classification system of the World Health Organization (International Classification of Diseases 11th Revision, ICD-11), GD also substitute the previous term “pathological gambling,” and was reclassified as an inclusion of “Disorders due to addictive behaviors,” while the previous classification in ICD-10 has not been completely removed, GD was also coded as an impulse control disorder.^[3]^ Studies showed that the prevalence of PG varies from 2.2% to 7% in PD population,^[4]^ while in normal population the percentage is 1.4%.^[5]^ Pharmacologic treatment of PD, mainly based on dopamine agonists (DAs), has been considered responsible for the development of impulse control disorders. In 2000, Molina et al^[6]^ firstly reported the association between antiparkinson drugs and PG, since then DAs have been linked to impulse control disorders by several clinical studies,^[7–10]^ and eventually 4 PD patients on piribedil were reported to develop PG in 2010,^[11]^ which aroused the attention of the academia for such dopamine agonist.

Piribedil is an orally active dopamine agonist that was marketed in 1960s,^[12]^ it has been widely used in the treatment of PD patients both as monotherapy and an adjunct to levodopa.^[13]^ Piribedil acts as a partial dopamine agonist of D2/D3, and α2 adrenoreceptors antagonist as well,^[14]^ such combination has been proved to be therapeutically beneficial in a number of aspects. Though some other dopamine agonists may perform broad-based and efficacious agonism efficacy, the specific partial activations of D2 and D3 receptors do not reduce antiparkinson efficacy of piribedil and may even offer advantages in several respects, such as reducing the incidence of dyskinetic movement or cognitive deterioration induced by hyperstimulation of dopaminergic receptors. When compared with other antiparkinson drugs with the properties of α2-AR antagonist, piribedil does not have any significant effects on 5-HT receptors, which might correspondingly reduce the risk of cardiovascular side-effects.^[15]^ Placebo-controlled trials have confirmed the utility of piribedil in controlling motor symptoms of early PD, and the safety of this non-ergot derivative is comparable to other dopamine agonists,^[12]^ nevertheless, the cases that associated piribedil with PG are worthy of our attention and further studies.

Herein we describe a PD patient with major depressive disorder who presented with low mood, insomnia, suicidal ideation, and PG accompanied by long-term use of piribedil.

## 2 Case presentation

A 28-year-old woman was sent to our ward by her family for the reason of being in low mood and suffering insomnia all night. We were informed that the patient had been addicted to network gambling with no obvious predisposing causes since the beginning of 2018. In August 2018, she began to be in depressed mood, and unwilling to communicate with other people. The patient always stayed up participating in online gambling, later she even stole her relatives’ credit cards, borrowed money from colleagues, or through mobile applications, but she still worked fine. In the next 6 months, the patient's condition got worse: she could not help gambling, and felt more anxious after her family found this secret. With the accumulation of the debt, her guilt got worse. However, she still could not kick the habit, and treated network gambling as a way to paralyze herself. The pace of her life began to become chaotic: she always cried all day and fear that other family members would abandon her, she refused to go outside or meet anyone, it seemed hard for her to deal with housework or take care of the children. After a fierce quarrel with her husband, the lady claimed that she did not want to live anymore though later there's no specific action. The family then felt it necessary to take her for systemic treatment and she scored 35 on the 17-item Hamilton Depression Rating Scale (HAMD-17). According to patient's history of present illness, what we considered the initial diagnosis is severe depressive episode without psychotic symptoms (ICD-10 F32.2). Venlafaxine, lorazepam, and buspirone were used for the relief of depression symptoms and the alleviation of her anxiety.

At the beginning we were confused and tried to find out if there were any psychogenic causes that might lead to her obsession with online gambling, a period of medical history caused our special attention: the lady claimed that she was diagnosed as Parkinson disease 5 years ago for the reason of left limb tremor, and kept taking piribedil (100 mg/d) since then, the effect was fairly ideal. By the way, we learned by further inquiry that the patient felt overexcited and satisfied when she gambled online, she knew it was not correct but it just impossible to resist the temptation. Every time she lost, the patient would feel upset and regretful, and might become irritable. She eventually lost her job, and the relationship with her husband was almost broken, which let her fall in deep despair. After careful consideration and analysis, we decide to revise the diagnosis as follows: severe depressive episode without psychotic symptoms (ICD-10 F32.2); mental and behavioral disorders due to multiple drug use and use of other psychoactive substances (ICD-10 F19). Considering the patient still in depressed mood accompanied by limb tremor symptoms and reeling gait (motor examination with the Unified Parkinson's Disease Rating Scale [UPDRS] revealed 18 scores), we made some adjustments on the medication: piribedil was discontinued with this hospitalization accordingly, venlafaxine was eventually replaced by bupropion, which the latter proved to be more tolerable, and benzhexol was added to control PD symptoms. We also consulted a neurological doctor, and were advised to add selegiline as a supplement. Three weeks later, the patient showed significant improvement in mood (HAMD-17: 11), and the PD symptoms were relieved (UPDRS motor: 7), she was no more obsessed in online gambling, and was discharged after careful assessment. During the 1-year follow-up, we learned that there was no recurrence of depressive episode or online gambling, the patient's family told us that she found a new job and her PD symptoms had been well-controlled during the past year.

## 3 Discussion

In this report we describe a PD patient receiving piribedil developed pathological gambling, and finally evolved into depressive disorder. After stopping piribedil and revising the choice of antidepressant, her obsession with online gambling and low mood were significantly relieved. We also formulated specific treatment plan and effectively controlled patient's recurring PD symptoms.

The patient in this case at first concealed her medical history of Parkinson disease and the truth that she had been taking piribedil for years, consequently, from the perspective of psychiatrics, we originally considered about the psychogenic causes of her addiction in network gambling, and analyzed the possible factors that might lead to PG such as family and peer influence, or other psychological and social factors, etc. In fact, pathological gamblers in some studies were reported more likely to show depression than nongamblers,^[1,16]^ and depression itself has been verified to be one of the non-motor symptoms of Parkinsons disease.^[17]^ Considering that patients in depression might treat gambling as an attempt to relieve negative mood states, there is no wonder that mood disorders have been frequently associated with pathological gambling.

Researchers tried to distinguish whether gambling disorder was primary or it was secondary to depression. McCormick et al^[18]^ found that 76% of patients in their gambling treatment program had major depressive disorder, among which there were 7 subjects reported that their depression seemed to precede the onset of the pathological gambling, but for most of the subjects, it seemed hard for them to tell the true chronological order between gambling and depressive episodes. An Epidemiologic Catchment Area (ECA) study conducted in St. Louis found that problem gamblers were at least 3 times as likely to meet criteria for major depression, schizophrenia, alcohol abuse, or anti-social personality disorder than nongamblers. By using age-of-onset information from a structured diagnostic instrument in this study, researchers confirmed that depression usually preceded gambling among problem gamblers.^[19]^ As a matter of fact, though there is a strong association between depression and gambling, it seems hard to reliably tell whether problem gambling resulted from depressive disorder, or depression was a natural consequence of gambling disorder. In the case mentioned above, the temporal relationship between pathological gambling and depressive episode was quite clear, we asked the patient and her family, they all denied there were any experience of depression before she got into online gambling.

With a period of treatment and observation, we adjusted antidepressant to bupropion, this atypical antidepressant exerts its effects mainly through inhibition of dopamine and norepinephrine reuptake,^[20]^ and has been proved to be clinically tolerable and safe for its lack of serotonin action, which as a result might not develop side effects such as drowsiness, weight gain, and sexual dysfunction.^[17]^ Given this, we made the adjustment in expectation of further control of PD symptoms and depression in patient. As early as in 1984, Goetz et al^[21]^ has confirmed the antiparkinsonian effect of bupropion in his study and recommended it as an adjunctive medication for PD. Later on there have been several clinical reports on effective treatment of depression with bupropion in PD patients,^[22–24]^ nevertheless, more clinical controlled studies are required to verify the efficacy of bupropion both for improvement in motor functions and remission of depression. Fortunately, with targeted use of antidepressant and psychotherapy, the patient's depressive symptoms have been significantly alleviated.

After acquiring the patient's medical history in detail, we began to focus on Parkinson disease and the widely used dopamine agonist, piribedil. The neurodegenerative process in PD was usually associated with nigral-striatal pathology and disruption in dopaminergic system,^[25]^ the DA therapy, however, can impact patients’ cognition and behaviors to some extent, which might lead to impulse control disorders, including pathological gambling.^[26]^ Angelo et al^[27]^ has discovered in a prospective observational study that the prevalence of impulse control disorder behaviors among PD patients can be quite stable (26.5%–29.3%) during a 2-year observational period, no matter patients received DAs or levodopa. Nevertheless, in another cross-sectional study conducted in the United States and Canada, 3090 subjects with treated idiopathic PD receiving routine clinical care were recruited, among which the prevalence of impulse control disorders in patients treated with DAs was 2.4 times of that in other subjects who did not received DA therapy (17.1% vs 6.9%).^[10]^ The mechanisms of PG or other impulse control disorder behaviors might be activation of D2 receptors in mesolimbic reward region, which is responsible for the loss of impulse control, while D3 receptors also have been frequently linked to gambling behavior.^[15]^ Therefore, as a partial dopamine agonist of D2 and D3, piribedil's side effect may even display in a relatively gentle way, that probably explain why there have not been a large amount of reports of piribedil causing PG.

A follow-up study has confirmed that after decreasing or discontinuing DAs, PD patients were reported experiencing varying degrees of relief from impulse control disorder behaviors.^[28]^ This study enlightened us that the dose reduction or withdrawal of DAs could represent a feasible strategy to reduce the occurrence of PG and its negative impact. But in clinical practice it is crucial to evaluate carefully for a balance between control of PG and the possible worsening of PD symptoms. In this case, the recurrence of limb tremor and gait instability after stopping piribedil warned us that we must pay attention to such balance, and treat Parkinson symptoms with caution. Benzhexol was used to control tremor symptoms, and selegiline, the monoamine oxidase type B (MAO-B) inhibitor, which proved to be efficacious in reducing motor fluctuations,^[29]^ was added after the consultation with a neurologist. As we expected, the patient's PD symptoms have been effectively relieved.

In summary, the above case reinforces the significance of screening for the emergence of pathological gambling and other impulse control disorders when commencing a patient on piribedil. The dose reduction and withdraw must be carried out under close clinical observation and evaluation.

## Author contributions

**Conceptualization:** Zhe Lu, Guanglei Xun.

**Project administration:** Guanglei Xun.

**Writing – original draft:** Yingtan Wang.

**Writing – review & editing:** Zhe Lu, Guanglei Xun.

## Abbreviations

Abbreviations: DAs = dopamine agonists, DSM = Diagnostic and Statistical Manual of Mental Disorders, GD = gambling disorder, HAMD-17 = 17-item Hamilton Depression Rating Scale, ICD = International Classification of Diseases, MAO-B = monoamine oxidase type B, PD = Parkinson disease, PG = pathological gambling, UPDRS = Unified Parkinson Disease Rating Scale.
